# Cannabis and Driving

**DOI:** 10.3389/fpsyt.2021.689444

**Published:** 2021-09-24

**Authors:** Godfrey D. Pearlson, Michael C. Stevens, Deepak Cyril D'Souza

**Affiliations:** ^1^Department of Psychiatry, Olin Neuropsychiatry Research Center, Institute of Living, Hartford Healthcare Corporation, Hartford, CT, United States; ^2^Department of Psychiatry, Yale University School of Medicine, New Haven, CT, United States; ^3^Department of Neuroscience, Yale University School of Medicine, New Haven, CT, United States

**Keywords:** cannabis use, driving impairment, motor vehicle driving, public health, roadside testing, THC, cannabinoids

## Abstract

As more states in the U.S legalize recreational and medicinal cannabis, rates of driving under the influence of this drug are increasing significantly. Aspects of this emerging public health issue potentially pit science against public policy. The authors believe that the legal cart is currently significantly ahead of the scientific horse. Issues such as detection procedures for cannabis-impaired drivers, and use of blood THC levels to gauge impairment, should rely heavily on current scientific knowledge. However, there are many, often unacknowledged research gaps in these and related areas, that need to be addressed in order provide a more coherent basis for public policies. This review focuses especially on those areas. In this article we review in a focused manner, current information linking cannabis to motor vehicle accidents and examine patterns of cannabis-impairment of driving related behaviors, their time courses, relationship to cannabis dose and THC blood levels, and compare cannabis and alcohol-impaired driving patterns directly. This review also delves into questions of alcohol-cannabis combinations and addresses the basis for of *per-se* limits in cannabis driving convictions. Finally, we distinguish between areas where research has provided clear answers to the above questions, areas that remain unclear, and make recommendations to fill gaps in current knowledge.

## Introduction

As increasing numbers of states in the USA legalize cannabis for medicinal and recreational purposes, the number of users is growing (1). Alongside this, the number of individuals operating motor vehicles under the influence of cannabis is necessarily also increasing. Since acute cannabis intoxication impairs some of the cognitive and psychomotor skills necessary for safe driver performance and decrements driving ability, the obvious concerns are the likely public health consequences for traffic safety of having more cannabis-intoxicated drivers on the road, and how to detect such drivers reliably. In turn this raises legal issues involving criminalization of cannabis-impaired driving. This raises the question of what strategies and procedures most reliably and validly detect cannabis-impaired drivers, in the extent of the knowledge base for making such decisions.

These topics are more complex to address than commonly assumed, and raise additional questions – not all of which have straightforward answers. Although these issues are examined in the following report, its intent is less be a comprehensive literature review, but rather more a systematic, critical exploration of the major questions in the field, their associated assumptions, and the extent to which current research has addressed (or not addressed) them. In those cases where these answers or adequate evidence to address important questions are still lacking, we point out the gaps in knowledge and suggest how they might be addressed. The major topics addressed are as follows:

- What is the epidemiologic evidence that cannabis is linked to motor vehicle crashes?
- How does the pharmacokinetic profile of THC differ from that of alcohol?
- What are epidemiologic trends in cannabis-related motor vehicle crashes?
- To what extent does cannabis impair driving-related behaviors and cognitive processes, which behaviors and abilities are most affected, and to what extent, following an acute cannabis dose?
- How do we assess cannabis-intoxicated drivers at the roadside?
- How valid is it use simulated driving research methods to make conclusions about the effect of cannabis on real-world driving ability? Does cannabis impair both virtual and actual on-road driving ability?
- How do the intoxication profiles of alcohol and THC differ in regard to driving impairment?
- Is cannabis' impairment related temporally to cannabis dose or to blood levels of THC or its metabolites?
- What is the time course of cannabis-related driving impairment?
- Can we detect cannabis-impaired drivers at the roadside reliably, and what is the validity of “*per se*” THC blood level limits in detecting cannabis-impaired drivers?
- Are alcohol/cannabis combinations more impairing (synergistic) than either substance used alone?

### Background: Cannabis Remains a Public Health Concern With Regard to Motor Vehicle Crashes

Motor vehicle accidents (MVAs) are among the top 10 leading causes of morbidity and mortality worldwide (2). In 2012, within the United States (US) there were about 33,561 fatal MVAs, in addition to 1,634,000 reported MVAs that caused injury (3). Fatal crashes were 32,166 by 2015 and 36,800 in 2018. Traffic crashes are amongst the leading cause of death in 5–34 year-olds (4), and are arguably preventable. While overall fatal crashes have remained stable or decreased over time, those due to drugged driving are trending up over time, from estimates in the US of 1,716 in 1993, to 6,612 in 2015 (5). Alcohol and cannabis are very important contributors to both impaired driving and MVA's (6). Aside from alcohol, cannabis is the primary drug detected in the US drugged driving cases and fatal motor vehicle crashes. But as we explore later, this statistic may be misleading due to the very marked persistence of THC in the body after consumption that is not necessarily reflective of impairment.

In the Fatality Analysis Reporting System (FARS) of the NHTSA, there were 8,617 reported crashes in 2012 involving drivers with a BAC ≥ 0.04, which resulted in 9,428 fatalities (3): in 2013 the NHTSA estimated that alcohol-impaired-driving fatalities accounted for 31% of motor vehicle crash (MVA) fatalities (7). In the National Survey on Drug Use and Health, cannabis was the most commonly used illicit drug in 2013 (8). Daily or almost daily use of cannabis increased from 5.1 to 8.1 million persons between 2005 to 2007, and 2013 (8). In 2013, 9.9 million persons and 40% of current illicit drug users admitted to driving under the influence of substances at least once in the past year (6). According to the FARS, in 2012, 2,083 reported MVAs occurred while driving under the influence of cannabis (DUIC) resulting in 2,208 fatalities (3). Many of these reliable figures came from research done nearly a decade ago. Now, with the increasing legalization and decriminalization of recreational cannabis and the legalization of medical cannabis in many states in the US the numerator in terms of more drivers being exposed to cannabis has increased. In addition there are long-term trends in cannabis available for public consumption, both a greater percentage of THC and increasing THC-to-CBD ratios (9). It is prudent to expect that as greater numbers of motor vehicle drivers are exposed to increasingly higher concentrations of THC, the likely trend is that more cannabis-related motor vehicle crashes will occur.

Next to alcohol, cannabis is the second most frequently found substance in the bodies of drivers involved in fatal MVAs. In Colorado, the proportion of drivers in fatal MVAs who were cannabis-positive increased from 5.9% in the first 6 (prior to the commercialization of cannabis) to 10% by the end of 2011 (post-commercialization) (10). Similarly, in Washington State, the average yearly percentage of DUIC cases positive for Delta-9-tetrahydrocannabinol (THC) and its principal metabolite THC-COOH increased from 19.1 and 27.9%, respectively, in 2009–2012 to 24.9 and 40.0%, respectively after the legalization of cannabis (11). Furthermore, while the prevalence of alcohol and other drugs in the same population of suspected impaired drivers submitted for testing did not change during this same 5 year period; cannabis was the only drug to increase in frequency (11). Interestingly, the proportion of cannabis-positive drivers involved in fatal MVAs has not changed in non-medical cannabis states (10). While this does not necessarily establish causality, it suggests that an increase in the use and acceptance of cannabis may be associated with DUIC. In Canada, DUIC within 1 h of cannabis use increased from 1.9% in 1996–7 to 4% in 2004 (12). In 2012, ~35% of all fatal MVAs involved either alcohol or cannabis, and when used together (BAC ≥ 0.04 and positive for cannabis) they accounted for 948 reported crashes and 1,025 fatalities (12). An important caveat to these data relates to the persistence of THC in the body long after the phase of acute intoxication has passed, an issue discussed below in the pharmacokinetics section.

While the effects of alcohol on driving are well-known and have been widely studied (13), the effects of cannabis or its constituent cannabinoids on driving are less clear (14), and even less is known about the effects of the combination of alcohol and cannabinoids on driving. While there are penalties to driving with a blood alcohol content (BAC) higher than 0.08%, there are not corresponding clear-cut limits to blood THC levels. Also, using simple formulas that take into account use the number of drinks consumed within a specified time frame, individuals can estimate their current BAC and therefore, make assumptions about whether it is legal for them to operate a motor vehicle. Reliable, corresponding information for cannabis is not available.

Before examining some of these and other surrounding issues in more detail, it is important to review briefly basic information that underpins many of the issues that we will discuss. This review takes place in the following two sections.

### What Is Driving?

Before looking in detail at cannabis' effects on driving, let's first ask a more basic question: “what is driving?” One way to consider this issue is to conceptualize driving as a pyramid of component behaviors and abilities, many of which are employed in other behavioral and cognitive contexts. A bottom-up view, beginning at the base of the conceptual pyramid, comprises specific constituent cognitive domains necessary for driving, starting with the least complex, such as simple visual perception and more habitual motor skills such as steering, that exists more at an operational level, and are located conceptually at the pyramid's base. As one ascends the pyramid, one travels through increasingly more complex domains such as visual reaction time, to higher-level tasks such as visual-motor integration, divided visual attention and visual working memory. Mid-level driving abilities such as car-following involve tactical skills. The most complex tasks such as overtaking, involving higher-level strategic skills are located toward the top of the pyramid, with driving itself as an emergent property, at the apex (15).

### Pharmacokinetics of THC Compared to Those of Ethanol

Many of the questions regarding the onset and duration of cannabis' impairing effects, the meaningfulness of detecting THC and its metabolites in biological samples relative to impaired driving and correlations between such levels and degree of impairment derive directly from knowledge of the pharmacokinetics of THC. Thus, a discussion of the facts regarding this topic is essential as a prelude to the following sections. And because so much conceptual confusion has arisen from attempts to equate the pharmacokinetics of THC with those of ethanol, a brief section contrasting the two is fruitful.

Ethanol in the form of beverage alcohol is extremely water-soluble. Because of this, alcohol can be easily diluted in aqueous solutions, so that spirits such as grain alcohol or high-proof vodka can be transmuted into the form of cocktails. Once imbibed, alcohol distributes to all physiological compartments quickly and evenly in predictable ways, since the human body is mostly composed of water. And thus biological samples from blood or breath (which contains high amounts of water) reflect both the amount of alcohol imbibed, and the amount present in the brain, which in turn reflects current levels of intoxication and impairment. Breath and blood alcohol concentrations can be straightforwardly measured (using a rather simple device, the “breathalyzer” in the case of breath) and breath alcohol concentrations (BrAC/BAC) can therefore be readily and quickly assessed at the roadside, indexing impairment. Because of ethanol's straightforward distribution in the body and fairly rapid, non-complex metabolism, BAC levels are proportional to ingested dose and decline predictably over several hours thereafter. The only complicating factor is gastric emptying, which can delay alcohol absorption when slowed, such as after eating fatty foods.

Almost none of these above facts apply to the pharmacokinetics of THC, the main intoxicating ingredient in cannabis (16, 17). As a separate issue, herbal cannabis itself is complex in several respects, containing not only THC but cannabidiol which can modulate THC's intoxicating effects, as well as various terpenes that may enhance THC intoxication or alter its passage across the blood brain barrier (18). The pulmonary route is extremely effective as a means of efficiently conveying THC or CBD to the bloodstream and hence to the brain. However, cannabis is administered in very different formulations and by various routes: orally as “edibles,” by smoking in cigarettes with or without tobacco, via tinctures oro-mucosally and from vaporizers that either evaporate cannabinoids from plant material, or use concentrated extracts of THC with or without other chemicals mixed with a vehicle, often in “vaping” devices such as pens. Each of these routes of administration and formulations is associated with different characteristic absorption patterns as regards rates and efficiency. And in common with alcohol, individual rates of metabolism vary with the extent (quantity, frequency) of use (16, 19).

Smoking and “vaping,” common routes of cannabis administration, are quick and efficient methods of delivering THC from the lungs to the brain. Slightly lower, but generally similar peak THC concentrations are achieved after smoking as compared to intravenous administration. Plasma THC levels are detectable almost immediately after the first cigarette or vape puff, with subjective and objective drug effects appearing shortly thereafter. Plasma THC concentrations increase rapidly, peaking at ~3–10 min after the final inhalation (16, 19). They then fall rapidly as the drug is absorbed and within about 20–30 min reach a low, relatively stable plateau that persists over several hours. THC-induced impairment on many measures declines slowly for ~5–6 h following acute dose in a manner that is generally unrelated to this post-peak THC blood level.

Oral absorption is slower and less efficient than with smoking, with a significantly more delayed onset of drug effect, and with intoxication that is then more sustained (20), with lower peak THC concentrations than those that follow smoking. Reasons for these differences include more variable absorption from the gut, gastric breakdown of THC, and significant first-pass metabolism in the liver to both psychoactive 11-OH-THC (that is more potent as an intoxicant than THC) and to inactive metabolites (21). The delay (~120 min) to reach peak concentration is significantly longer than with smoking. Inhaled THC is often referred to as having an average bioavailability of around 30% (17, 22), although it had a systemic bioavailability of ~50% in a recent, carefully controlled study using protocol-based inhalation of vapor, compared to estimates from other studies of ~6% for oral dosing (23). It should be noted that these estimates are only approximate, since there is also substantial variability e.g. in how different individuals smoke cannabis cigarettes, e.g. in terms of amount and depth of inhalation.

Rather than being hydrophilic like alcohol, THC is extremely lipophilic. It distributes quickly into organs with higher blood supplies including the brain, heart and liver, moving later into body areas with less perfusion. Because of its fat solubility, it leaches into, and persists in body regions with high fat content, including the brain and adipose tissues. With chronic use, significant accumulation in these latter tissues can occur with gradual release, even if cannabis is not smoked for a period of time. This release and redistribution can lead to its subsequent metabolism and detection in bio-samples including urine days to weeks following last cannabis use. THC is metabolized primarily in the liver and excreted in the urine and feces.

Because its absorption, distribution and metabolism differ so markedly from that of alcohol, the relationship between plasma THC and intoxication is also both different and more complex than that of ethanol levels and intoxication. The concentration of THC in brain and in plasma are dissociated in time, so that by the time intoxication is beginning to ramp up, the plasma peak of THC is already long past. Plasma levels do not clearly reflect dose once the plasma peak has subsided. Intoxication too, is less dose-related than with alcohol, and peak THC blood levels are not clearly related to subsequent maximal levels of behavioral impairment. In contrast, as we noted above, with alcohol peak blood and breath alcohol levels correspond closely in time and are proportional to peak levels of intoxication and drug-related impairment.

Because breath is moist and does not contain lipids, there is almost no available THC present; the number of molecules of the compound is in the picogram range and an extremely sensitive technology is necessary to detect it. All of these factors pose multiple problems for law enforcement personnel attempting to link the presence and amount of THC in blood to recency of use and to the degree of impairment in motor vehicle drivers who may be operating under the influence of cannabis. This difficulty is further amplified when considering the significant lag between intercepting such drivers and obtaining blood specimens in which to measure THC concentration.

### What Is the Epidemiologic Evidence That Cannabis Is Linked to Motor Vehicle Crashes?

An important part of the evidence that cannabis impairs motor vehicle driving and consequently leads to more motor vehicle crashes and deaths relies on epidemiologic reports. While the annual number of fatal vehicle crashes in the US is trending down in recent years (in part due to more consistent enforcement of regulations and higher penalties for drunk driving), the number of motor vehicle crashes involving positive THC tests has increased (24). As summarized by McCartney et al. (25), these data derive from two main sources. The first is the numbers of motor vehicle crash drivers who are found post-crash to have THC or other cannabis metabolites in their blood. The second source derives from epidemiologic trends in motor vehicle crashes in those states that have legalized or decriminalized cannabis consumption, compared to those that have not.

Rogeberg and Elvik's (25) meta-analyses (25–27) looked at data derived from ~240,000 individuals across multiple published studies, investigating the association between acute cannabis consumption and an individual either being responsible for or being involved in a motor vehicle crash. The overall odds ratio showed a low- to-moderate magnitude, but significant risk, with the OR for such involvement being 1.36. For comparison, that number is much less than that for alcohol, where the OR is ~20 at a BAC of 0.10, as estimated by the same authors. Other estimates e.g., Biecheler et al. (28), provide ORs of 2.3 for cannabis alone, 9.4 for alcohol alone, and 14.1 for cannabis and alcohol in combination.

Annual patterns of excess traffic fatalities due to cannabis were examined by Kamer (29) who quantified changes in traffic for mortality rates from 2008 in Alaska, Oregon, Washington and Colorado compared to control states that had not legalized cannabis. These authors documented increased fatality rates in Alaska and Oregon and initial increases followed by decreases in Washington and Colorado. Their overall conclusion was that approximately double excess deaths in the USA occurred per billion vehicle miles traveled due to cannabis intoxication. Both the Kamer study and a separate investigation by Aydelotte (30) agreed that an approximate doubling of excess motor vehicle related deaths occurs attributable to cannabis. If accurate, this statistic translates into cannabis being involved in ~18.6% of overall US motor vehicle deaths, equivalent to an additional 6,800 individuals involved traffic fatalities (based on the official estimate of ~36,800 in 2018).

There are methodologic caveats applicable to both of the above-mentioned approaches. What's unknown, yet germane is when these drivers had consumed cannabis relative to the indexed MVA. This question is important because as noted above, THC and several of its metabolites can persist in blood and body tissues for days-to-weeks following acute use. Thus, detection of THC or one of its metabolites does not necessarily equate to current intoxication. Also not always recorded is what percentage of the presumed cannabis-impaired drivers also had alcohol or other driving-impairing substances in their blood, even if these were below the legal cutoffs for intoxication. As we explore later, few experiments that have examined the synergistic effects of acute cannabis exposure concomitant with legally permissible levels of blood alcohol. If the two substances are synergistic in their ability to impaired driving, then quantifying both is clearly important.

There are also some methodologic problems in tracking temporal patterns of motor vehicle crashes or traffic fatalities following cannabis legalization in a particular state, compared to states that did not legalize. One is that the date of the enabling legislation does not align well with availability of cannabis in the legalizing state. From the time point that the legislation is passed, to customers being able to buy cannabis from dispensaries may vary from months to years, a factor which needs to be taken into account. In addition, would-be purchasers may be able to cross state lines from a non-legal to a legal state in order to make purchases, interfering with a researcher's ability to make accurate relative cross-state comparisons.

### Does Cannabis Impair Driving–Related Behaviors and Cognitive Processes?

The weight of evidence from many epidemiologic studies, studies of chronic cannabis smoking, and laboratory studies of the consequences of acute dosing, strongly support that cannabis use deleteriously affects driving-related cognitive test performance on a variety of tasks conceptually linked to motor vehicle driving. Relevant data on acute dose effects are summarized in Table 1 through 3 below. Meta-analytic studies summarize acute cannabis-provoked impairment affecting multiple domains relevant to vehicle operation (84) Table 1 details these acute cannabis effects on driving-related cognitive tasks. Table 2 lists studies that have examined actual driving behaviors, mainly in simulated or on-road driving, whereas Table 3 summarizes this information relative to the three major driving skill levels detailed in the “what is driving?” section above.

**Table 1:** Cannabis impairment effects on driving-related cognitive tasks.

Useful Field of View (31–37)
Motor Pursuit/Tracking (32, 38–50)
Time Estimation or Self-Paced timing (51–58)
Distance Estimation [57^*^, 61^*^]
Set shifting/Task switching (59)
Working Memory/Executive functioning (37, 49, 60–62)
Serial Addition/Subtraction (63)
Hand/Body Steadiness/Coordination (38, 39, 45–48, 64–66)
Choice Reaction Time (33, 40, 45, 46, 63, 65, 67–69)
Short-term Memory (61, 70–77)
State dependent learning (78)
Vigilance, signal detection (33, 47, 79)
Visual Search [36^*^, 62^*^, 73^*^]
Information processing speed [34~, 67~, 77, 84, 85]
Maze Accuracy (80)
Danger perception/Risk taking [5, 36^*^, 50, 61^*^, 87–90]
Stress/distraction Susceptibility (47, 81)
Attentional Allocation (EEG) (82, 83)

* *Indicates that results were cannabis dose dependent*.

~ *Indicates that data were collected during actual vehicle driving*.

**Table 2:** Impairment of driving behaviors by cannabis.

**Driving measure**	**Cannabis effect**
Fewer Fine Manipulative Steering Movements/Steering Instability	No effect (34, 50, 85, 86)
Increased Steering Wheel Reversals/Variability	No effect (34, 74, 87)
Increased Speed Variance/Excessive Speed or Slowness	No effect (34, 49, 61, 86, 88–90)
Decreased Cornering Stability, Speed Variability on Curves	(41, 89)
Increased Braking Distance/Stop Time	(41, 49, 61, 86)
Increased Lateral Position Errors, Variability, or Lane Deviation	(74, 88, 89, 91–94)
Increased Collisions, Decreased Time to Collision, or Slowness Avoiding Other Vehicles or obstacles	No effect (49, 88–91, 93)
Errors in Speedometer Tracking	(86)
Altered Passing Behavior	(58, 88, 95)
Increased Start Time (in response to light signal)	(57, 61)

*“No effect” indicates that the behavior was measured but no cannabis-related impairment was detected*.

**Table 3:** Driving scenarios and key outcome measures for 3 hierarchical driving tasks.

**Name**	**Conventional outcome measures**	**Cannabis-related impairment**	**Exploratory time-locked outcome measures**	**Attention/control manipulation**
Road Tracking Task (Operational)	1 – Standard Deviation of Lane Position (SDLP)	(69, 89–94)	Corrections when SLDP ≥1 SD from participant's mean	Unpredictable lateral wind gusts
Car Following Task (Tactical)	1- Coherence, 2- Modulus, and 3- Delay signal analysis indices	(91, 96)	Lead car peak acceleration or deceleration	High rates (g) of lead vehicle speed change
Gap Acceptance Task (Strategic)	1- Size of gap chosen 2- Minimum time-to-contact (TTC)	(97)	Onset of the acceleration through chosen traffic gap	Cross-traffic in opposing directions

Three major inter-related questions derive from consideration of these data. 1. What is the evidence linking the listed domains in Table 1 to actual impaired on-road driving, as opposed to theoretical impairment? 2. How useful are available neurocognitive tests for detecting recent cannabis use? 3. How do we best use this informational foundation to guide research that seeks to identify field sobriety tests which can (a) accurately detect drug-induced cognitive impairments and/or (b) predict risky driving? These questions are addressed in subsequent sections. Notably, cannabis-induced changes on a computer-based critical tracking task significantly correlated to altered tactical vehicle tracking behavior during on-road driving (98).

### Driving-Relevant Cognitive Tasks That Were Sensitive to Cannabis-Related Impairment in Previous Studies

#### Key to Table 1

The above studies were conducted using a wide variety of dosing routes, doses of administered cannabis and volunteer subject types as regards prior experience with cannabis. Experimental designs varied widely, and impairment within each category was measured using a wide selection of metrics. This variability makes both comparisons across studies and drawing of generalized conclusions difficult. However, the first three metrics (useful field of view, motor pursuit tracking and time estimation), showed robust impairment in multiple studies across a fairly wide variety of experimental circumstances.

### Does Cannabis Impair Actual Driving Behaviors?

If So, Which Behaviors, to What Extent and for How Long After an Acute Cannabis Dose?

Table 2 Quantitative measurements of actual driving behavior under either real on-road or simulated driving conditions.

Many of the behaviors were assessed since they are impaired in alcohol-intoxicated drivers.

Table 3 lists examples of translating driving measures altered by cannabis derived from Table 2 into standard outcome measures for simulated driving tasks of ascending complexity. For example, standard deviation of lane position (SDLP) is a measure of lateral position and lane deviation, elicited at a simple, operational level of driving complexity that in this example involves the subject needing to continue driving in a straight line while dealing with unpredictable lateral wind gusts necessitating vehicle correction by steering.

Delays during the car following task incorporate aspects of speed variance and stopping time during more complex tactical driving maneuvers. The task involves the subject maintaining a fixed distance from a lead vehicle that slows down or speeds up unpredictably.

Gap acceptance choice and time to contact measures incorporate measures of slowness in avoiding other vehicles and altered passing behavior cited in Table 2 during the execution of passing maneuvers, during a complex, strategic-level gap acceptance task. This task involves the subject making the decision when to safely pass a stalled vehicle, necessitating lane change under conditions of variable oncoming traffic.

#### How Does One Assess Driving Impairment Validly and Reliably?

We describe four separate approaches to answering this question. The most direct way to address this issue is to have research subjects drive a real vehicle on a real road, while acutely intoxicated on cannabis (91). Although this procedure is the gold standard, it is subject to practical and ethical constraints. These include interaction with other on-road vehicles, and the impossibility of enacting certain scenarios (e.g., animal runs onto the road unexpectedly, or a leading car brakes suddenly). As an alternative, a closely-related approach to deal with this set of problems has been to employ real vehicles on closed-course experimental highways such as Virginia Tech's Smart Roads, a set of state-of-the-art, closed test-bed research facilities closely resembling real highways, managed by Virginia Tech Transportation Institute (VTTI) in cooperation with the Virginia Department of Transportation (VDOT)^1^ On some of these test roads a series of sensors embedded in the tarmac communicate with computer equipment located inside the test vehicles. Dual-operator controls such as those used in driver education vehicles are available in case of emergencies when the intoxicated subject exhibits dangerous driving. In the case of alcohol, intoxicated driving research using an instrumented vehicle on a simulated test highway (99) has been performed using the Smart Road, and revealed generally similar deficits to those exhibited on a desktop driving simulator.

A third approach is to recognize that under most circumstances, one cannot ethically or practically allow research subjects to drive a real vehicle on a real road. Instead, one can use an extremely high-end driving simulator that can accommodate the chassis and controls from a variety of real vehicles, such as that used in the National Advanced Driving Simulator (NADS) at Iowa, that has been used to assess cannabis-intoxicated driving (100). The NADS is unique in incorporating sufficient technology to provide highly realistic, real-time kinesthetic feedback that closely mimics that of real driving, and an extremely wide and realistic field-of-view. All three of the above approaches score most highly on face validity, but entail various practical hurdles such as being relatively difficult and/or expensive to access.

A fourth approach, and therefore the most practical solution, resembles the set up immediately above, but in a more affordable, lower-tech incarnation. This translates to in-lab testing with sufficient construct/criterion validity to provide useful data. For many investigators, this involves the use of driving simulators, that range anywhere from videogame-like apparatuses linked to a typical desktop -sized computer display screen, steering wheel and gas/brake pedals at one end of the spectrum, to an actual, repurposed, instrumented motor vehicle chassis on a motion base (to provide some form of kinesthetic feedback), situated in front of a wall-sized projection screen (to provide greater field-of-view), at the more sophisticated extreme. The advantages of such setups are obvious: subjects can be intoxicated with the study drug/placebo in the lab and subsequently asked to drive in a number of pre-programmed scenarios to quantify their degree of impairment.

Simulators in general provide a controlled, safe environment that theoretically translates into real-world driving performance. A large number of scenarios can be pre-programmed in order to test driving ability under a wide variety of conditions, and these can be varied sufficiently to avoid learning effects. With driving simulators one can mimic scenarios that are unethical or impractical to test in real life, such as abruptly-appearing road hazards, weather changes, or similar unexpected scenarios. Furthermore, because of the ease of manipulating the environment, driving scenarios can be easily constructed that would be unsafe or impossible to create on a real roadway. We and others have shown that intoxicated driving under the influence of alcohol compares fairly closely to driving a real vehicle on a real, instrumented road (99), demonstrating validity. Major considerations for simulated driving include the degree of realism and sophistication (and therefore expense) of the relevant hardware, software, driving tasks and measurement capabilities. And underpinning these is the issue of validity, that is the extent to which simulated driving behavior can be used to draw inferences regarding the behavior of real-world highway driving in relevant, representative situations (for example in heavy traffic).

#### Does Cannabis' Impairment Profile in Terms of Domains Impacted and the Severity of Impairment Resemble That of Alcohol?

As summarized in Table 4 opposite, while both alcohol and cannabis impair aspects of driving behavior, the two drugs affect driving rather differently, with overlap in deficits mainly for weaving, and possibly for divided attention (although this latter is not well-studied for cannabis). Studies can only point toward generalities in the population, as there might always be exceptions of cannabis-intoxicated people who do not drive more slowly or carefully. Factors including youth, driving experience and substance tolerance may all influence the individual's response to a drug (14). It is worth emphasizing that few studies have directly compared driving impairment due to the two drugs in a head-to-head fashion, and almost none-in a design involving substantial numbers of the same subjects and assessments over a range of doses of both substances. Thus, any conclusions have to be tentative at this point. The conclusions summarized in the Table 4 are based in part on published work from our own laboratory involving simulated driving and subjects' self-reports, that has involved BAC levels ranging from 0.05 to 0.08 for alcohol (59, 87, 101, 110, 111) and more recent unpublished data (102) involving inhalation of vaporized doses of cannabis ranging from ~42.5 to ~65 mg. One major behavioral difference that we observe in our subjects is that cannabis-intoxicated volunteers report not only being aware of their likely driving impairment (see later section on impairment duration), but also overestimate its degree, and consequently tend to drive more slowly in an attempt to compensate for deficits. In contrast, alcohol-intoxicated subjects at a BAC of 0.08% or above are not only more likely to fail to recognize their actual impairment but are also more inclined to make impulsive behavioral choices, and at BAC's equal to or exceeding 0.1, to engage in dangerous driving behaviors such as driving at excessive speed, especially in risky situations such as when navigating their vehicle around curves.

**Table 4:** Contrasting alcohol vs. cannabis effects on simulated and actual driving behavior and associated cognitions.

**Impairment domain**	**Alcohol**	**Cannabis**
Awareness of deficit	Impaired (14)	Unimpaired (14)
Ability to compensate for deficits	Absent (14)	Present/partially present (14)
Tracking/lane position	Impaired [(101)^*^, (102)]	Impaired (100, 103, 104)
Divided attention	Impaired (87, 105)	Impaired (84)
Concentration	Impaired (81)	Impaired (84)
Reaction time	Increased (106)	Increased/No Change (106, 107)
Impulsive/risky choice making	Impaired (56, 108)	Unimpaired (56)
Excessive driving Speed	Present (109)	Absent (100)

* *Indicates dose dependent*.

Experiments that have examined brain responses to intoxicated driving, although few in number, also speak to different alterations provoked by the two drugs. As mentioned elsewhere in this article, Hartman et al.'s (100) simulated driving study using the NADS directly compared the two drugs in the same set of individuals. While cannabis only affected weaving behavior (measured by standard deviation of lane position/SDLP), alcohol impaired SDLP in addition to measures of lane departures and maximum acceleration.

### Are Alcohol/Cannabis Combinations More Impairing (Synergistic) Than Either Substance Used Alone?

This is important public health question, particularly if “safe” levels of the two substances that do not individually significantly impact driving, have a meaningful impact on decrementing driving behavior when combined. As Dubois et al. (112) note, in the realm of motor vehicle crashes the phenomenon of simultaneous combined alcohol/cannabis intoxication is on the increase, with a 5-fold increase in crashes involving detection of combined THC/alcohol from below 2% in 1991 to above 10% in 2008.

Simulated driving studies that have examined the nature of interactions between cannabis and alcohol are notably inconsistent in detecting synergy between the two substances vs. a purely additive effect, as noted by Hartman et al. (100). For example, Ronen et al. (113) reported that while there were no significant alterations in lane position variability when either 13 mg THC or 0.05% (BAC) alcohol were administered alone, the combination produced a significant increase in weaving behavior. Lenne et al. (69) reported significant independent main effects of both cannabis and alcohol, but found that the combination was purely additive without interaction/synergy. In an on-road study combining different THC doses with a 0.04% target BAC (an alcohol concentration considered insufficient by itself to produce behavioral change), the combination significantly increased SDLP (91). In Hartman et al.'s (100) double-blind, placebo-controlled study, both cannabis and alcohol were individually significantly associated with impaired lateral control (weaving) assessed by measures of SDLP. While cannabis only affected SDLP, alcohol impaired this measure as well as lane departures and maximum acceleration. In terms of equivalence between the two substances, while lower doses of cannabis administered through vaporization yielding 8.2 μg/L blood THC were associated with SDLP abnormalities similar to breath alcohol (BrAC) values of 0.05% (~0.05%, SDLP at 13.1 μg/L THC approximated 0.08% BrAC. Combining alcohol and cannabis in this study produced an additive rather than a synergistic effect on SDLP, with no interaction. The authors also noted that these THC concentrations collected during driving in their study were generally higher than those collected typically hours later by law enforcement in traffic-stop situations.

Epidemiologic studies also shed some light on this question Dubois et al. (112) examined combined THC/alcohol crash culpability in fatal car crashes. The study confined itself mainly to victims with a low levels of BAC of 0.08% or less. The authors estimated that each 0.01 BAC unit increased the culpability odds (COs) of a crash by ~9–11%. Drivers who were positive for THC alone had a 16% increase in COs, while combined THC/alcohol COs were synergistic, exceeding CO values for alcohol or THC alone. The authors stress that further research would be needed to clarify more specifically interactions between cannabis and alcohol concentration levels and driving impairment.

A reasonable overall conclusion from examining the above studies is that while there are many suggestions of a synergistic decrement in driving behavior – particularly for SDLP – when cannabis and alcohol are used together, there is also credible contradictory evidence arguing only for an additive effect. More importantly perhaps, most of the above studies demonstrate that there is a lack of comprehensive investigations exploring the full range of interactions across a variety of both BAC and cannabis doses/blood levels conducted in the same subjects to allow more meaningful comparisons. For example, investigators willing to repeat the rigorous design of the Hartman et al. (100) study across such an expanded range of doses of the two substances would provide a more definitive answer to this important question of synergy. So evidence is lacking to make solid conclusions at this time.

### Is Cannabis' Impairment of Driving Related Temporally to Administered Dose or to Blood Levels of THC or Its Metabolites?

Typical of experiments describing a generally poor correlational relationship between performance disruption and serum THC is the study of Ramaekers et.al. (98) who described effects in in 20 cannabis users smoking placebo or doses of 17.5 mg or 35 mg of THC per 70 kg body weight, and subsequently evaluating THC blood levels and various behaviors critical tracking task/perceptual motor control, motor impulsivity (using a stop signal task) and executive function (using the Tower of London paradigm) from 15 min to 5 h post drug challenge. As noted, their findings are in sharp contrast with those reported for alcohol intoxication, where behavioral disruption and BAC track closely. While legislators may wish for data showing straightforward relationships between blood THC levels and driving impairment that parallel those of alcohol, the widely different pharmacokinetic properties of the two substances, leading to a rapid fall in THC levels to a relatively steady, low baseline within ~20 min of an inhaled dose make this goal unrealistic.

A final consideration is that even if a candidate behavioral/cognitive task or biological measure (such as plasma THC) is sensitive to recent cannabis exposure, it may nevertheless be unrelated to on-road driving ability, and thus not useful as an index of fitness to drive.

### What Is the Duration of Cannabis-Related Driving Impairment?

Cannabis' peak impairment on driving performance is evident 20–40 min following inhalation (14), even as THC blood levels are long past their peak and continuing to diminish. By 1–2.5 h post-inhalation, behavioral impairment is still present but already beginning to diminish (14, 114). Because of this fairly time-limited impairment, several sources suggest that following acute use, cannabis consumers should wait a minimum of 3–4 h before attempting to drive (115). A recent paper from Arkell et al. (116) used a double-blind, within-participant randomized clinical trial with an active THC dose of 13.75 mg consumed by inhalation following vaporization, and measurements of driving performance in an actual vehicle on a real road. The major findings were that weaving (assessed by standard deviation of lane position/SDLP) was significantly greater at 40–100 min, but not at 240–300 min post-dose. Subjects' self-rated confidence to drive safely tracked poorly with actual measured SDLP, with participants significantly rating themselves as more impaired 4–5 h following active THC compared to placebo, despite SDLP being unimpaired by that time. It is important to note that consumption of “edibles,” with delayed onset and greater persistence of intoxication effects, and dosing via smoking or vaporization at a higher dose than used by Arkell et al. (116) would likely result in a greater period of impairment. Lastly, cannabidiol (CBD) administered simultaneously in vaporized cannabis does not significantly diminish THC-induced driving impairment (117), despite the fact that there is some evidence that CBD may alter either the pharmacokinetics (PK) of THC or modulate behavioral effects of the latter (118, 119). Recently Liu et al. (119) developed population PK models of THC and CBD. When high-dose CBD was inhaled at the same time as THC, the systemic availability of the latter decreased significantly. Interestingly, in the same set of experiments, frequent users of cannabis appeared to have higher systemic availability of both THC and CBD when high-dose CBD was administered.

It is worth drawing attention to the fact that the majority of driving studies have been performed on inhaled cannabis in younger subjects, and there is a paucity of studies on driving performance following oral administration of the drug, where there is likely to be increased variability of both onset and duration of impairment. In addition, despite increasing use of cannabis in individuals aged over 60 (typically to help manage insomnia and chronically painful conditions), there are very few studies quantifying cannabis-related driving impairment in such older individuals following any route of drug administration, although likely age-related alterations in pharmacokinetics are likely.

### Can We Detect Cannabis-Impaired Drivers at the Roadside?

A number of recent papers have surveyed various issues pertaining to roadside detection of putatively cannabis-impaired drivers (120–123). Because roadside detection of alcohol impaired drivers works so well and straightforwardly, this model has undoubtedly biased expectations, procedures, expectations and policies in the case of cannabis. However, we will present evidence that these guiding assumptions fail to carry over from one substance to the other.

How does roadside detection of cannabis-impaired drivers unfold in the real world? Typically, law enforcement personnel will either stop a driver for “probable cause” (that in practice could constitute anything from an observation of vehicle weaving, to a non-functional taillight), or detain them at a random police checkpoint. If the driver appears to be impaired, or cannabis-related paraphernalia is visible within the vehicle for example, then law enforcement personnel will generally administer a battery of roadside tests for impairment detection. If these are abnormal, they will assess the subject's BrAC via a “breathalyzer” device. If the breathalyzer reading is negative, then the police may request that the subject's blood be drawn for drug testing at nearby facility. The average time between the police pulling over such a driver and the blood sample actually being collected in this manner is 90 min (124). It is important to note that roadside tests of driver impairment/intoxication were originally developed for detecting alcohol-impaired drivers, and the extent to which they are applicable to cannabis impairment has not been rigorously examined. For example, common test items that validly screen for alcohol-intoxicated drivers include measurements of postural sway, nystagmus, heel-to-toe walking and repeating a sentence correctly. Many if not all of these items are minimally impaired by cannabis intoxication (14). Similarly, while drug recognition expert's (DREs) are consistently reliable in identifying alcohol-impaired drivers, they are more variable in their ability to correctly identify cannabis-impaired individuals (125–127). A number of current experiments are underway to find the most reliable ways to assess individuals driving under the influence of drugs (DUID) including cannabis.

This raises the issue of whether there are available other, more feasible candidate screens for roadside testing of cannabis-impaired drivers. Ideally, such a test must be simple, quick, and sufficiently robust to test in real-world situations, for example by roadside at night in a situation where there is perhaps little light and noisy traffic passing by. Such a test must be practical to administer at the roadside, e.g., on a tablet computer, must demonstrate accurate prediction (acceptable false-positive/false-negative rates), and have a narrow confidence interval, high reproducibility, generalizability, and acceptable face validity. Ideally it should also display strong criterion-related validity. Several such candidate measures are currently undergoing testing. These include Milburn's DRUID test battery (128–130), assessments of postural instability using electronic devices, measurements of brain state using portable EEG devices (131), pupillary responses to flashes of light, laptop-based cognitive test batteries, and hand-held, instrument-based cognitive testing devices, such as the Intoximeter^2^. It should be emphasized that all of these investigations are preliminary, and no valid, reliable screening paradigm is yet available. Moreover, with any such potentially useful approach there is a need to validate it against a valid and reliable measure of impaired driving, in terms of determining its relevance, then subsequently to conduct extensive field trials.

Other issues with roadside detection of cannabis-impaired drivers include dual or multi-intoxication, for example the individual as consumed small amounts of both cannabis and alcohol which are acting synergistically, mentioned above. It would be useful to know whether one can identify deficits specific to cannabis, or either mimicked by or potentiated by other drugs of abuse (or alcohol). Another potential difficulty is the lack of personal baseline information for police from an individual being tested at the roadside. This presupposes the presence of a large behavioral database for a particular task, normed to age and sex as appropriate. One possibility is that a useful test for screening for cannabis-impaired drivers could involve capitalizing on combinatorial batteries, where several deficits detected are unlikely to co-occur by chance, yielding a “fingerprint” of cannabis impairment.

In the real world, policy determinations might need to choose between (1) detection of recency of use or (2) tests whose results accurately predict driving impairment. There are potential new developments in biological measurements of THC at the roadside that are relevant to this discussion. As mentioned earlier, one major problem with presumptively intoxicated driver testing involves the lag between a driver being examined by police on suspicion of driving under the influence of drugs and the relevant blood sample being obtained. This deficiency is potentially addressed by a “THC breathalyzer” device currently under development or by specific field sobriety tests for cannabis behavioral impairment that reflect impaired driving. The device manufactured by Hound Laboratories (Oakland, CA), that is currently undergoing field testing and validation, is touted as a cannabis “Breathalyzer,” that aims to detect trace amounts of THC from cannabis smoked in last 2–3 h. The technology is based on the fact that very small amounts of THC can purportedly be found in exhaled breath up to 3 h after one last inhaled cannabis. Because these quantities are tiny (picograms) as THC is not water-soluble, any successful detection technology has to be ultra-sensitive. If trials of the device are encouraging, it subsequent employment will at least address the current pronounced lag between police roadside testing and THC measurement, but does not fully address in itself the other problems noted above, i.e., does recency of smoking cannabis equate to impaired driving in the individual being tested. The difficulties in addressing the latter approach to find tests sensitive to actual driving impairment are exemplified by the legal issues surrounding “*per se*” laws.

### What Is the Status of “*per se*” Laws for Cannabis-Impaired Driving?

A number of authors have examined issues of biological specimen collection to detect cannabis intoxicated drivers (132, 133). Wong et al. (134) usefully distinguish between three different approaches to identify cannabis-impaired drivers. The first is “effect based” requiring proof that the drug impaired the defendant's driving. This approach pertains in most US states, but its enforcement is complicated by two factors: proving that the drug resulted in impairment, and a paucity of agreed-on and standardized methods to quantify drug-induced driving (135, 136) impairment. If there is no consensus in how to measure driving impairment, attempts to link any type of predictive test to driving becomes problematic. The second approach consists of legislating that any detectable amount of THC or a metabolite is sufficient to convict the driver of drugged driving (137–139). The obvious difficulty with this approach concerns the well-documented lengthy persistence of THC and its metabolites in blood and to some extent oral fluids, particularly in regular users. Some investigators have shown that THC in stays in the body for many days, even up to a month after last use, obviously well-after the period of acute driving-related behavioral impairment (103, 140, 141).

The third approach is the use of “*per se* limits,” as adopted by several US states. The intent of such legislative efforts is to set a quantitative threshold for blood THC concentrations that is reliably associated with driving impairment, and thus constitutes an offense “*per se*” (i.e., in and of itself). In part this assumption derives from (or is a supposedly logical extension of), well-established associations between blood alcohol concentrations and driver impairment. In the case of THC, the presumption in establishing such a threshold is that a defined range of blood or saliva THC concentrations exists that reliably separates cannabis-impaired drivers from those who may have residual detectable amounts, but are unimpaired (137–139, 142). Thus, *per se* cannabis DUI laws create a new traffic safety violation defined by state-defined levels of THC or its metabolites, where exceeding this legal limit by itself serves as proof of impairment (62, 137).

Many investigators in the field believe that the current evidence supporting such threshold is slim and that such legislative efforts are premature (138, 143). *Per se* laws vary enormously from state to state in the US. For example, 13 states prohibit driving with any amount of detectable plasma THC, while a handful of states specify a legal THC cutoff level, above which driving is illegal. These cutoff values themselves are also not consistent. In CO, MT, IL and WA, they are set at 5 ng/ml of blood (or in some cases, such as IL, blood breath or urine); in NV and OH the value is 2 ng/ml. The remainder of states prohibit driving while “incapacitated by” or ”under the influence of“ cannabis, so-called “effect-based DUI laws” as mentioned above, which essentially rely on a subjective judgment. While each such state hews to a slightly different legal standard, both these latter definitions translate to an ill-defined prohibition on “driving while high.”

In 2007, an international group of experts met to determine whether a *per se* THC threshold could reasonably be set (138). They concluded that “…a THC concentration in the serum of 7–10 ng/ml is correlated with an impairment comparable to that caused by a blood alcohol concentration (BAC) of 0.05%. Thus, a suitable numerical limit for THC in serum may fall in that range…. (and)…. offers an empirical basis for a *per se* limit for THC that allows identification of drivers impaired by cannabis. The limited epidemiological data render this limit preliminary.” Further evidence from a variety of sources has cast this initial conclusion in doubt. The essential problem is that because of the distinct pharmacokinetics of THC, leading to a persistence of the drug and its metabolites in blood, and enormous inter-individual variability in metabolism of THC, the establishment of *per-se* limits is much more complex and ill-defined than for alcohol. The worst-case scenarios yield either false positives, resulting in conviction for driving under the influence of drugs (DUID) based on cannabis that the subject may have consumed days to weeks ago, when they are now completely unimpaired, or conversely false negative cases, where an individual's driving is in fact impaired by recently-consumed cannabis, but their THC blood or saliva level is below the *per se* threshold.

Recent publications shed considerable light on these concerns. Logan (143) examined data from 2 sources in ~ 600 drivers arrested for DUI in which only THC was present compared to ~ 350 drug-free controls, examined by a drug recognition expert, and ~4,800 drivers arrested for DUI who tested positive for THC or its metabolites. The key findings were that compared to drug-free controls, the arrestees performed more poorly in psycho-physical tests including the Standardized Field Sobriety Test, but that the finger-to-nose test was the only indicator for which performance differed according to where the subjects were in the >5 ng/ml or <5 ng/ml THC group (with the former showing more errors). Analysis of alternative cut points ranging from 1 to 10 ng/ml failed to identify any threshold THC level that was useful as a limit and would provide an acceptable level of agreement with the SFST. The authors reported that all of the candidate THC concentration thresholds would have misclassified a substantial number of drivers, producing both large numbers of false positives and false negatives, and concluded that “based on this analysis, a quantitative threshold for *per se* laws for THC following cannabis use cannot be scientifically supported.”

Similarly, a 2019 report issued by the Congressional Research Service (144): concluded that “Research studies have been unable to consistently correlate levels of cannabis consumption, or THC in a person's body, and levels of impairment. Thus, some researchers, and the National Highway Traffic Safety Administration, have observed that using a measure of THC as evidence of a driver's impairment is not supported by scientific evidence to date.” Finally, a recent study by Arkell et al. (145) concluded that “The blood and oral fluid *per se* limits examined often failed to discriminate between impaired and unimpaired drivers,” …. “Moreover, blood and oral fluid THC concentrations were poorly correlated with driving impairment. … It is almost impossible to infer how much cannabis was consumed, or when it was consumed, based solely on a given concentration of THC in any biological matrix.”…. “Due to erratic and route-dependent differences in THC pharmacokinetics as well as significant inter- and intra-individual variability, blood and oral fluid THC concentrations, unlike BAC (blood alcohol concentrations) for alcohol, provide little information as to the amount of cannabis consumed or the extent to which an individual may be intoxicated. Collectively, these results suggest that the *per se* limits examined here do not reliably represent thresholds for impaired driving.”

A final issue is that people who use cannabis regularly may well-develop measurable tolerance to intoxicating and impairing effects of the drug. Although those effects remain to be established for driving performance, they would not translate legally into such individuals being allowed to drive with higher blood THC levels, paralleling the legal status with regard to alcohol tolerance. A separate issue is that the individuals who use cannabis daily or more frequently (e.g., to treat an ongoing medical condition), may always exceed the *per se* limit. This is another challenge that would likely need to be overcome if *per se* laws were to be widely adopted.

In summary, current evidence from the above studies suggests that efforts to establish *per se* limits for cannabis-impaired drivers based on blood THC values are still premature at this time. Considerably more evidence is needed before we can have an equivalent “BAC for THC.” The particular pharmacokinetics of cannabis and its variable impairing effects on driving ability currently seem to argue that defining a standardized *per se* limit for THC will be a very difficult goal to achieve. Furthermore, there has been virtually no testing of driver impairment following oral consumption of “edibles,” with virtually all testing being performed on inhaled cannabis derived from flower or vaporized liquid, despite the increasing consumption of cannabis in edible forms, and the distinct pharmacokinetic difference in time of onset and duration of intoxication between the oral vs. inhaled dosing methods.

## Discussion

It should be clear from the various studies reviewed in this paper, that cannabis-impaired driving is a real public health problem, in that it results in such drivers being significantly more likely to be involved in motor vehicle crashes (134). This is the case despite widespread emerging agreement that the relative risk of such impaired driving is significantly lower than other legislated drug use while driving, such as that resulting from alcohol or cocaine (25). However, the issue posed regarding cannabis legalization is not whether we intend to substitute one drug (cannabis) for another (e.g., alcohol), but that as a society we are deciding whether to legalize a new, previously illegal substance and thereby expose new individuals to the drug's side effects alongside its putative benefits. With increasing legalization of cannabis and therefore rising use rates/availability of the drug, particularly in forms containing higher percentages of THC, it is a mathematical certainty that this problem of cannabis-impaired driving will worsen. It is not possible to predict at this point whether absolute rates of cannabis-involved motor vehicle crashes will approach those seen with other substances, although the numbers of drivers projected to be cannabis users over the next decade will certainly increase significantly. So it would be mistake to conclude the problem of cannabis-intoxicated driving should not be addressed.

Given this context however, a number of conceptual and practical difficulties attend the reliable understanding and detection of such driving impairments, not all of which are widely recognized. Thus, while the intent of legislative efforts to detect and sanction cannabis-impaired driving are well-intended, their execution often falls short. In part this is because of the lack of understanding of the limitations of what the relevant science does and does not support.

What can we conclude to date regarding cannabis-impaired driving, based on available research? We know that the pharmacokinetics of alcohol and cannabis are distinctly different (17), as are for the most part the cognitive/behavioral domains relevant to impaired driving affected by each substance (14). These differences must be properly appreciated and recognized to prevent an unfounded, yet common tendency to elide the two drugs in matters pertaining to time courses of impairment, allocating significance to biological detection of the drug's presence or concentration in bodily fluids or exhaled breath, and developing roadside impairment testing or screening batteries. In terms of an affordable experimental laboratory paradigm with which to quantify impaired driving following acute dosing with cannabis, simulated driving appears overall to be sufficiently valid and reliable to be a reasonable surrogate for on-road driving experiments (100). Investigators have a reasonable idea of the duration of driving impairment following moderate doses of inhaled cannabis (146). We can be moderately confident of these observations tempered by the relatively small numbers of well-controlled studies (and small numbers of participants within those studies) examining cannabis-impaired drivers. Such small-scale studies are necessary because of the complex pharmacokinetics of THC compared alcohol, necessitating rather complicated and necessarily expensive experiments, and their downside is noted below.

As a general point, the literature also suggests that the issue of cannabis-impaired driving is bedeviled by a number of issues. What then are some of these difficulties and unknowns? It is important for both scientists and legislators to identify questions that either have the potential to direct this field forward, or that are needed to prevent it from running astray. In a climate where policymakers are particularly keen to legalize cannabis (amongst other reasons to enhance state revenues), there is appropriate consequent pressure to enact legislation to detect and deal with cannabis intoxicated drivers. This urgency however can lead to development of laws that are insufficiently reliant on the relevant known science, and that make unwarranted assumptions such as inappropriately adopting approaches that are appropriate for alcohol-intoxicated drivers, but not so for cannabis-intoxicated ones.

Thus, there remain many uncertainties and open questions regarding cannabis-intoxicated driving. Some such difficulties include lack of clarity in interpreting body fluid sampling after fatal and non-fatal crashes (the lengthy persistence of THC in the body after an acute dose makes interpretation complex) (140). As a consequence of the more complex pharmacokinetics of THC compared to that of alcohol, there is no straightforward way at the present time to equate measurements of THC levels in blood or saliva and current driving impairment. Related to this issue, there are still open questions regarding the time course of THC-related driving impairment following different acute doses of certain forms of the drug. In particular, duration and characteristics of impairment following increasingly-used cannabis concentrates with very high THC content (“dabs,” “shatter” etc) and of edibles at different doses are understudied.

Unlike screening for alcohol impairment, as yet there are no agreed-on reliable and valid roadside sobriety testing paradigms for cannabis-impaired drivers, a lack of agreed-on norms for such testing (124), and approaches to account for a lack of sober baseline testing on presumptively impaired individuals (For example an older driver may be clumsy or exhibit mild psychomotor slowing at their sober baseline; this may yield a false positive on a poorly designed screening test).

Epidemiologic studies are often necessarily uninformative as to specific doses of THC that subjects consumed prior to driving, the drivers' impairment in key psychomotor domains at the time of the crash or assessment by law enforcement, and their biological levels of cannabinoids at the time of incapacitation as opposed to those a significant time later. While experimental laboratory studies can be informative regarding some of these questions, as they are under direct control, such investigations are complex to carry out, labor-intensive, usually expensive and notably difficult to obtain appropriate approval for. For all of those reasons they are often characterized by low subject numbers and thus statistically underpowered as noted above. In addition they lack direct validity, as few of them are conducted in real vehicles on an actual highway. Simulated driving in general is a safe valid substitute approach to test drug-related driving impairment, alongside cognitive & behavioral tests. However, the extent to which behaviors on different types of driving simulators are valid surrogates for on-road driving is underexplored.

Because of their vastly different pharmacokinetics, roadside testing measures and blood drug levels for cannabis impairment are not comparable to those available for alcohol (125), nor as simple to interpret. And because blood THC is still the gold standard, the practical difficulties in obtaining blood sampling and the time lag involved greatly complicate this issue. Standardization of collection times would be greatly desirable. Devices currently under testing that claim to detect the presence of smoked cannabis in breath samples may hopefully provide a reliable index of recent cannabis use, although this does not necessarily equate either to dose consumed or level of intoxication/impairment. Partly as a consequence of the above, and partly due to the complex pharmacokinetics of THC, *per-se* laws as currently construed are based on insufficient information and need to be more data-driven than they are at present (144, 145). The risk from this lack of knowledge is bi-directional: it can result in both under-detection of genuinely cannabis-impaired drivers and unnecessary criminal conviction of individuals with detectable THC in physiological samples who are nevertheless no longer intoxicated or driving-impaired.

A final significantly understudied question is the issue of synergy between impairing effects of alcohol and cannabis, particularly in light of their frequent simultaneous consumption. A particular concern is that low doses of each drug in combination, where neither alone is sufficient to cause manifest driving impairment, will lead to such impairment (147). If such synergy exists (and it is not yet convincingly demonstrated), then the characteristics of such combined intoxication need to be studied and defined as a first step to identifying them so that they can be reliably screened for and detected at the roadside.

## Recommendations

Cannabis-impaired driving is an under-appreciated risk, and one with growing public health consequences. The situation is complicated by the somewhat skewed, agenda-driven reporting of this area of inquiry. For example both proponents and opponents of cannabis legalization each interpret statistical reports of motor vehicle crashes in relationship to cannabis legalization differently, hoping that the data can help further their own agenda. Relying on established science can definitely help the debate, particularly in instances where science finds itself bumping up against public policy, with legislators and others needing to be more current/topical about the existing research, so that they can make the best, most informed, policy decisions.

Looking first at the public health issue, because cannabis-intoxicated individuals are relatively aware of their impairment, particularly in comparison to alcohol-intoxicated drivers, many cannabis users erroneously assume that they are therefore safe to drive. Public service announcements emphasizing risks of “stoned driving,” such as those used in Australia, would be a useful investment in the US. And although the evidence for synergy of impairment between alcohol and cannabis is still preliminary, this point could be easily incorporated into such PSA's, at least as a means of raising awareness of a potential problem. In the interim though, more research needs to be conducted in this area, given its potential public health importance.

Until there is more evidence-based consensus of opinion on meaningful thresholds for *per se* laws, we would recommend against reliance on such legislation. This is particularly the case given the significant inconsistencies in threshold values currently determined by different states in the US, and the rather weak scientific basis for such decisions. Any such laws cannot claim to be strongly based on current scientific evidence, which suggest collectively that standard based on detectable blood THC levels are not useful. These relatively recently ascertained facts tend to contradict established legislative efforts to demarcate cut offs. A related issue is the still current disconnect between demonstrating the presence of THC in a physiological sample taken from a putatively intoxicated driver and the assumption of driving impairment.

There is widespread agreement on the dearth of available valid roadside tests that assess cannabis-related behavioral patterns specifically, and an obvious need to develop such screening paradigms that index actual cannabis-related driving impairment, rather than mere intoxication that may be unrelated to such impairment. It is important therefore to first validate experimentally any such putative field sobriety impairment measures in the context of concomitant on-road or simulated driving.

Finally, because cannabis concentrates and edible forms of the drug are becoming more popular (148, 149), and are both potent sources of THC and little-studied in terms of their types and time courses of driving impairment, it would be prudent for the National Institute on Drug Abuse to devote more resources on studying the effects of these forms of cannabis, and developing procedures for making them available to investigators for this purpose.

## Author Contributions

GP, MS, and DD contributed to writing the manuscript. GP and DD conducted the literature search. All authors contributed to the article and approved the submitted version.

## Funding

GP and MS are holders of Research Contract DTNH2216R00036, from the National Highway and Traffic Safety Administration, and of research award R01 DA 038807, from the National Institute on Drug Abuse, both of which study aspects of cannabis-intoxicated driving. DD NIAAA: 1R21AA024257, to study alcohol/cannabis interactions on driving.

## Conflict of Interest

The authors declare that the research was conducted in the absence of any commercial or financial relationships that could be construed as a potential conflict of interest.

## Publisher's Note

All claims expressed in this article are solely those of the authors and do not necessarily represent those of their affiliated organizations, or those of the publisher, the editors and the reviewers. Any product that may be evaluated in this article, or claim that may be made by its manufacturer, is not guaranteed or endorsed by the publisher.

## Footnotes

^1^ https://www.vtti.vt.edu/facilities/virginia-smart-roads.html

^2^ https://www.intox.com/?keyword_session_id=vt~adwords%7Ckt~%7Cmt~b%7Cta~442241803803&_vsrefdom=wordstream
